# CLIMATE CHANGE: California’s 2020 Vision

**DOI:** 10.1289/ehp.117-a103

**Published:** 2009-03

**Authors:** Graeme Stemp-Morlock

As the source of 1.4% of the world’s greenhouse gas (GHG) emissions and 6.2% of U.S. emissions, California is a significant actor on the global climate stage. In the coming decades, if current scientific thinking proves correct, California and many other parts of the world could face climatic fallout in the form of rising sea levels, more wildfires, water shortages, and pollution-aggravating heat waves. These changes, in turn, could incur immense public health costs and other economic losses. Against this backdrop, the Golden State has adopted a comprehensive plan—unprecedented in scale and scope—designed to reduce GHG emissions substantially by 2020. The long-range goal is to achieve emissions that are 80% below 1990 levels by 2050.

On 11 December 2008, the state’s Air Resources Board (ARB), a department of the California Environmental Protection Agency (CalEPA) and the agency responsible for protecting air quality, approved the “Climate Change Scoping Plan” for reducing the state’s carbon footprint. The plan, developed by the ARB in coordination with representatives from other agencies, aims to reduce GHG emissions back to 1990 levels over the next 11 years; this means cutting emissions by about 15% from today’s “business-as-usual” levels. The ARB’s scientific staff predicts the plan’s measures will reduce the state’s GHG annual emissions by a total of 174 million metric tons. Development of the plan was a central requirement of AB 32, the state’s Global Warming Solutions Act of 2006.

Pivotal to the plan is a cap-and-trade program that will cover 85% of the state’s GHG emissions and give industries a financial incentive to reduce those emissions. By setting a clear, mandatory cap on emissions, the plan allows the market itself to identify the most cost-effective ways to achieve that limit. Tradable emission “allowances” or permits are then distributed in an amount that equals the total emissions permitted by the cap. Complementing the cap-and-trade program will be a mix of strategies that combine technology-forcing performance standards, fees, and other policies.

The California program is designed to work with the Western Climate Initiative (WCI), a group of states and provinces working collaboratively to reduce GHG levels on a regional scale. Rather than give allowances away for free, the WCI plans to auction at least 10% of permits at the outset while allowing other states to auction more if they choose. California plans on eventually auctioning all permits, perhaps as soon as 2016. “These auction revenues could net anywhere from one to five billion dollars or more annually by 2020, helping to fund the transition to a ‘green economy,’” says Bernadette del Chiaro, an energy analyst for the advocacy group Environment California.

Other key features of the California plan include a requirement for utilities to generate one-third of their electricity from solar, wind, and geothermal sources by 2020; implementation of standards for low-carbon fuels, cleaner cars and trucks, and energy- and resource-efficient construction; and full deployment of the Million Solar Roofs Initiative, an incentive program aimed at installing 3,000 megawatts’ worth of new rooftop solar capacity by 2017. The complete plan is available at http://www.arb.ca.gov/cc/scopingplan/document/psp.pdf.

“We believe that market structures like cap-and-trade are essential for businesses to meet their obligations until green technology comes to market,” adds Amisha Patel, policy advocate for climate change and energy issues for the California Chamber of Commerce. However, the plan has not received universal praise. The 12 December 2008 *Los Angeles Times* quoted James Duran, chairman of legislative affairs for the California Hispanic Chambers of Commerce, calling the plan “an economic train wreck waiting to happen” as far as small businesses go.

The regulations and legislation tried out in California will likely be watched closely by the new White House. “What we do here will resonate and will have repercussions on the kinds of approaches and programs that the Obama administration will work to implement,” says ARB spokesman Stanley Young. “We are very conscious of the leadership role that we’ve got, and we want to develop a program that will integrate well with any federal program.”

